# Electrochemical Approach for Isolation of Chitin from the Skeleton of the Black Coral *Cirrhipathes* sp. (Antipatharia)

**DOI:** 10.3390/md18060297

**Published:** 2020-06-02

**Authors:** Krzysztof Nowacki, Izabela Stępniak, Enrico Langer, Mikhail Tsurkan, Marcin Wysokowski, Iaroslav Petrenko, Yuliya Khrunyk, Andriy Fursov, Marzia Bo, Giorgio Bavestrello, Yvonne Joseph, Hermann Ehrlich

**Affiliations:** 1 Faculty of Chemical Technology, Institute of Chemistry and Technical Electrochemistry, Poznan University of Technology, ul. Berdychowo 4, 60965 Poznan, Poland; 2 Institute of Semiconductors and Microsystems, TU Dresden, 01062 Dresden, Germany; enrico.langer@tu-dresden.de; 3 Leibniz Institute of Polymer Research Dresden, 01069 Dresden, Germany; tsurkan@ipfdd.de; 4 Faculty of Chemical Technology, Institute of Chemical Technology and Engineering, Poznan University of Technology, Berdychowo 4, 60965 Poznan, Poland; marcin.wysokowski@put.poznan.pl; 5 Institute of Electronics and Sensor Materials, TU Bergakademie Freiberg, Gustav-Zeuner str. 3, 09599 Freiberg, Germany; iaroslavpetrenko@gmail.com (I.P.); andriyfur@gmail.com (A.F.); yvonne.joseph@esm.tu-freiberg.de (Y.J.); 6 Department of Heat Treatment and Physics of Metal, Ural Federal University, Mira Str. 19, Ekaterinburg 620002, Russia; juliakhrunyk@yahoo.co.uk; 7 The Institute of High Temperature Electrochemistry of the Ural Branch of the Russian Academy of Sciences, Akademicheskaya Str. 20, Ekaterinburg 620990, Russia; 8 Dipartimento di Scienze della Terra, dell’Ambiente e della Vita, Università degli Studi di Genova, Corso Europa 26, 16132 Genova, Italy; marzia.bo@unige.it (M.B.); giorgio.bavestrello@unige.it (G.B.); 9 Center for Advanced Technology, Adam Mickiewicz University, 61614 Poznan, Poland

**Keywords:** chitin, biological materials, electrolysis, Antipatharia, black corals, *Cirrhipathes* sp.

## Abstract

The development of novel and effective methods for the isolation of chitin, which remains one of the fundamental aminopolysaccharides within skeletal structures of diverse marine invertebrates, is still relevant. In contrast to numerous studies on chitin extraction from crustaceans, mollusks and sponges, there are only a few reports concerning its isolation from corals, and especially black corals (Antipatharia). In this work, we report the stepwise isolation and identification of chitin from *Cirrhipathes* sp. (Antipatharia, Antipathidae) for the first time. The proposed method, aiming at the extraction of the chitinous scaffold from the skeleton of black coral species, combined a well-known chemical treatment with in situ electrolysis, using a concentrated Na_2_SO_4_ aqueous solution as the electrolyte. This novel method allows the isolation of α-chitin in the form of a microporous membrane-like material. Moreover, the extracted chitinous scaffold, with a well-preserved, unique pore distribution, has been extracted in an astoundingly short time (12 h) compared to the earlier reported attempts at chitin isolation from Antipatharia corals.

## 1. Introduction

Chitin is composed of β-(1,4)-*N*-acetyl-d-glucosamine units, and plays a crucial role in the formation of skeletal structures in invertebrate organisms, where rigidity and strength are required [1]. This most abundant aminopolysaccharide has been isolated and identified in skeletal structures of diverse species of fungi, algae and invertebrates (i.e., sponges, hydrozoans, mollusks, worms, insects, spiders and crustaceans) [2,3,4,5,6,7,8,9,10,11,12]. Here, chitin is present in the form of biocomposites, being chemically bound to proteins, pigments and other polysaccharides, as well as mineral phases [13,14]. Consequently, its extraction from such biocomposites is fraught with a number of methodological difficulties that must be overcome using different approaches. 

Nowadays, chitin of marine invertebrate origin is commonly isolated via two types of extraction process: chemical or biological [15,16]. In brief, the chemical treatment requires three main steps, namely deproteinization, demineralization and depigmentation. The deproteinization is generally carried out as the first stage. During this part of the process, the chitin-based structure is treated with alkaline solutions, which causes the dissolution of most of the proteins. This step is highly important in terms of medical and technological applications, because it determines the purity of the obtained product as well as the deacetylation degree and the possible hydrolysis of the chitin polymeric chain, depending on temperature conditions used [17]. The demineralization step usually follows hydrolysis of the proteins of the chitinous structure, and involves its treatment with acid solution (i.e., CH_3_COOH or HCl). This step is conducted to treat highly mineralized biomaterials; it ensures the elimination of calcium carbonates via decomposition of these insoluble compounds into water-soluble calcium salts, along with the release of carbon dioxide [13]. The third step, called depigmentation, is a treatment optimally carried out by adding highly reactive oxidizing agents, such as hydrogen peroxide. However, all three steps of the chemical treatment rely on extraction agents that have to be used in great excess, thus generating effluents that are hazardous to the environment. Moreover, a relatively long treatment time, along with the increased temperature of the deproteinization process, can cause uncontrolled degradation of the chitinous polymeric chain [18,19,20]. The biological isolation of chitin, being an alternative method to the chemical treatment, uses microorganisms which produce diluted organic acids and enzymes to fulfill the role of the chemical extraction agents. Despite the longer time of treatment, chitin obtained via the biologically catalyzed process possesses a better-preserved spatial structure than that of an industrial source [21]. Regardless, in order to increase efficiency and reduce the environmental impact of chemical the process, novel and modified methods have been developed [22,23,24]. The recently reported assisted methods are mostly focused on the use of microwave irradiation as the accelerating factor [9]. Indeed, the application of this approach leads to a significant reduction of treatment time (from days to a few hours) [15]. 

Among the assisted methods for the isolation of chitin, electrolysis has been relatively poorly investigated. Only one proposed approach that includes the electrochemical treatment of chitin-containing crustacean exoskeletons has been described to date [25,26,27]. The principle of this method is based on the electrolysis of a diluted NaCl aqueous solution to ensure the acidic and alkali treatment of the crustacean *Gammarus pulex*’s (Linnaeus, 1758) biomass. However, being based on the electrolysis of a low-concentration NaCl solution, this method is characterized by significant treatment time (13–19 h) [28]. The evolution of chlorine gas on the anode surface, which is a highly corrosive compound, is another drawback of the method [29]. Thus, electrolysis-assisted isolation of chitin has not received much attention over traditionally used extraction procedures. As we recently showed with experiments on chitin from the skeleton of the marine sponge *Aplysina aerophoba* (Nardo, 1833), the electrolysis process can be very flexible in terms of electrochemical conditions [16]. Thus, there is possible scope for the improvement of this overlooked approach—for example, by the application of different electrolytes.

**Figure 1:**
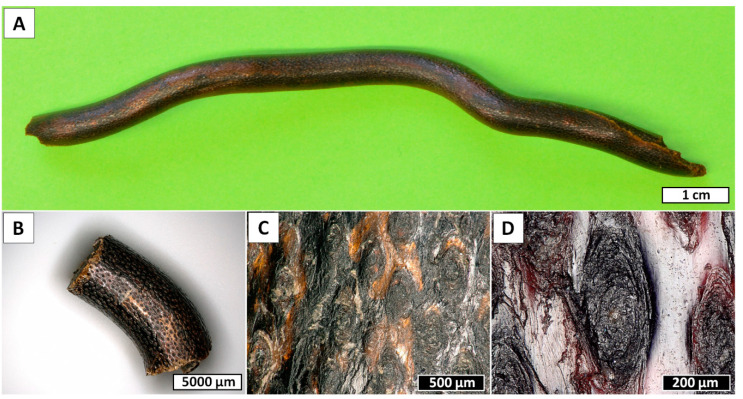
Overview of the *Cirrhipathes* sp. coral fragments used in the study. (**A**) Central portion of the unbranched, unpinnulated stem of the colony. (**B**) Close-up view of the skeletal surface showing the multiple longitudinal rows of spines. (**C**) Basal plates of the spines after erosion. (**D**) Close-up view of one spine basal plate showing the concentric layers of skeleton.

**Figure 2:**
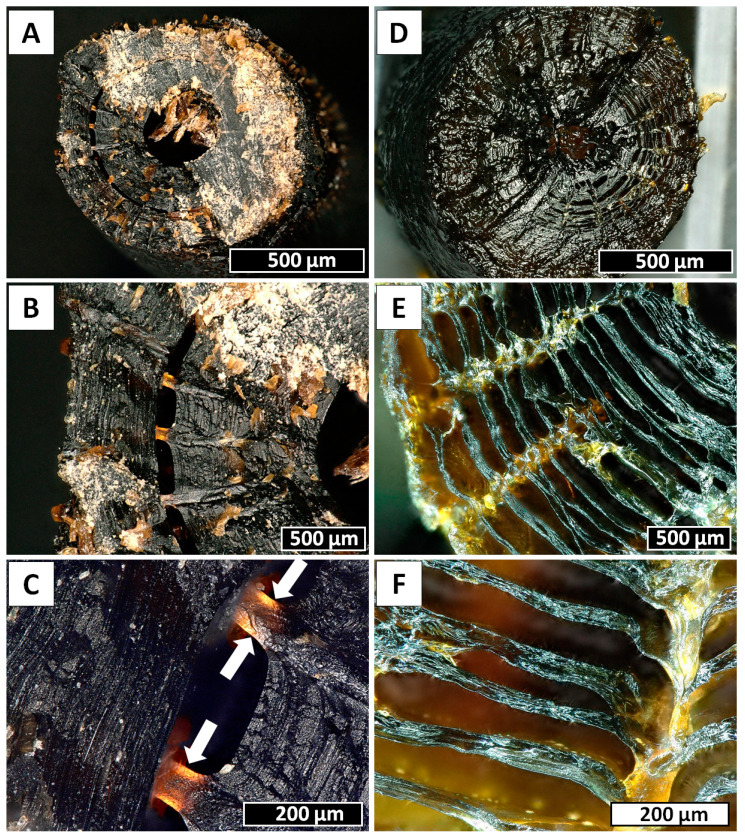
Insights into the inner structure of *Cirrhipathes* sp. skeleton. (**A**) Transversal section of the stem, showing a clearly hollow central canal surrounded by concentric layers of skeleton. The outer surface is covered in small triangular spines. (**B**,**C**) Spines’ roots visible between the skeletal concentric layers. (**D**) Transversal section of the stem with a central canal partially closed by a skeletal septum. (**E**,**F**) Clusters of concentric skeletal layers intersecting perpendicularly with the spines’ roots, connecting vertically the outer surface with the internal channel.

In this study, we present the results of electrochemical isolation of chitin from the black coral *Cirrhipathes* sp. (Antipatharia, Antipathidae) (Figure 1 and Figure 2) for the first time. The intense research on these marine invertebrates has been focused mostly on the isolation and characterization of the special, dark-pigmented biopolymer antipathin, and the accompanying diphenol compounds [30,31,32]. Unfortunately, *Cirrhipathes* species are poorly described as a source of chitinous scaffolds. Previously, we have shown that the isolation of chitin from selected black corals is mostly performed via the chemical method [33]. It involves long, alternating alkaline and acidic extraction steps, and hence the duration of treatment often exceeds 7 days [33]. Therefore, this method can be modified in order to reduce the treatment time and amount of chemicals used. For this purpose, we decided to modify the known electrolysis method for the isolation of chitin from crustaceans, as reported earlier [34]. Since the described process is based on similar steps to the chemical process, it was essential to ensure a highly alkaline environment in order to achieve a successful result. Therefore, in this study, a concentrated Na_2_SO_4_ aqueous solution was utilized as the electrolyte, and a novel electrochemical method (which combined a well-known chemical treatment with in situ electrolysis) was investigated in terms of its usefulness for the extraction of chitinous scaffold from *Cirrhipathes* sp. skeletons. 

## 2. Results

**Figure 3:**
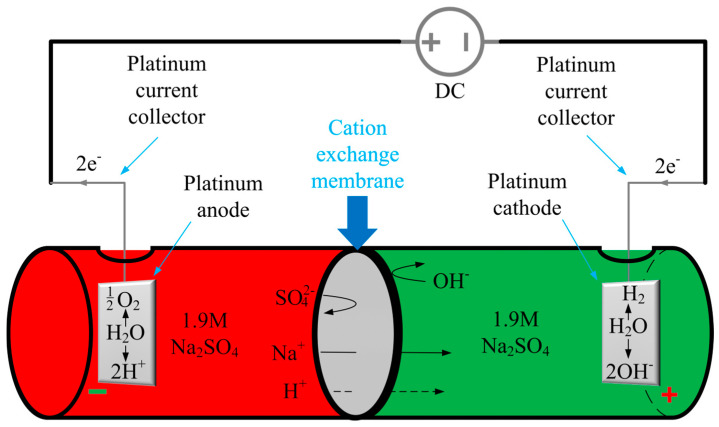
Schematic illustration of the electrolysis cell assembled in this study, and a general principle of Na_2_SO_4_ aqueous solution electrolysis [44].

The water electrolysis process is a well-known electrochemical phenomenon that has to be thermodynamically forced via the flow of a direct electric current from an external source [35]. In order to pass the current between two electrodes, a specific electrolytic cell (electrolyzer) must be constructed. Briefly, the modern electrolyzer is composed of two symmetrical, polarizable electrodes, made of electrically and chemically inert materials with high active surface areas. Usually, both electrodes are dipped in an electrically conductive solution (electrolyte) and separated with an ion exchange membrane (cation, anion or bipolar), forming two compartments [36,37,38,39,40,41]. The chamber with the anode contains the electrolyte solution, called the anolyte, whereas the chamber with the cathode is filled with the catholyte. Aqueous solutions of low molecular salts, such as NaCl or Na_2_SO_4_, which are generated as by-products in a wide variety of chemical processes, serve as perfect substrates for the production of alkalis and acids by electrolysis. The splitting of the Na_2_SO_4_ aqueous solution into NaOH and H_2_SO_4_ solutions, which occurs in the cation exchange membrane (CEM) of the electrolyzer, is one of the most popular ways to utilize the overproduction of this salt [42]. Figure 3 shows the basic principle of this process [43]. During Na_2_SO_4_ aqueous solution electrolysis (decomposition of water particles), fundamental electrochemical reactions take place on the electrodes’ surfaces. These redox reactions result in an excess of H^+^ and OH^−^ ions in the anolyte and catholyte, respectively. Simultaneously, sodium ions from the anolyte migrate through the CEM towards the cathode, where they are reduced to a sodium metal, which immediately reacts with the water to form NaOH [44]. Thanks to this phenomenon, it is possible to establish and change the pH in each part of the electrolyzer by applying a specific potential. This feature can be useful in terms of the extraction process that is responsive to the pH parameter. Thus, we have applied the fundamental feature of chitin, i.e., strong resistance to alkaline solutions up to temperatures between 70 °C and 80 °C, where its de-acetylation and transformation into chitosan occurs [45]. However, in our method, the alkaline environment has been achieved via electrolysis.

**Figure 4:**
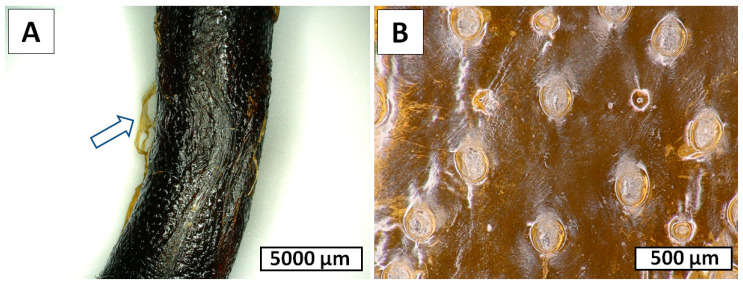
Rejection of the membranous film-like structure following 3 h of catholyte treatment on the *Cirrhipathes* sp. black coral surface (**A**) becomes well visible. This biological material was still pigmented and kept regular spine formations (**B**) on its surface (See also Figure 5).

**Figure 5:**
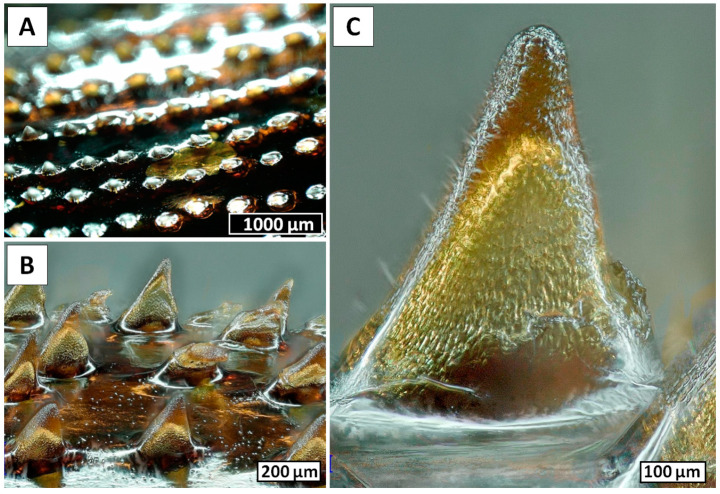
Regular longitudinal rows of spines (**A**,**B**), which are characteristic of the surface of *Cirrhipathes* sp. skeleton, remain without visible changes (**C**) also on the surface of the rejected film-like membrane after 3 h of electrolysis. The surface ornamentation of small, sparse papillae is visible.

The changes in the surface morphology of the selected fragments of *Cirrhipathes* sp., occurring during the electrolysis within the electrolytic chamber, were monitored using digital microscopy. Nearly three hours after the electrolysis treatment was started, the rejection of the upper layer, in the form of a membrane-like film, became observable (Figure 4A,B). At this step, no structural changes on the surface of this film could be observed at the microlevel (Figure 5), however, the fact of the appearance of such an alkali-resistant structure implies the destruction of proteins that secured it in the coral’s skeleton.

**Figure 6:**
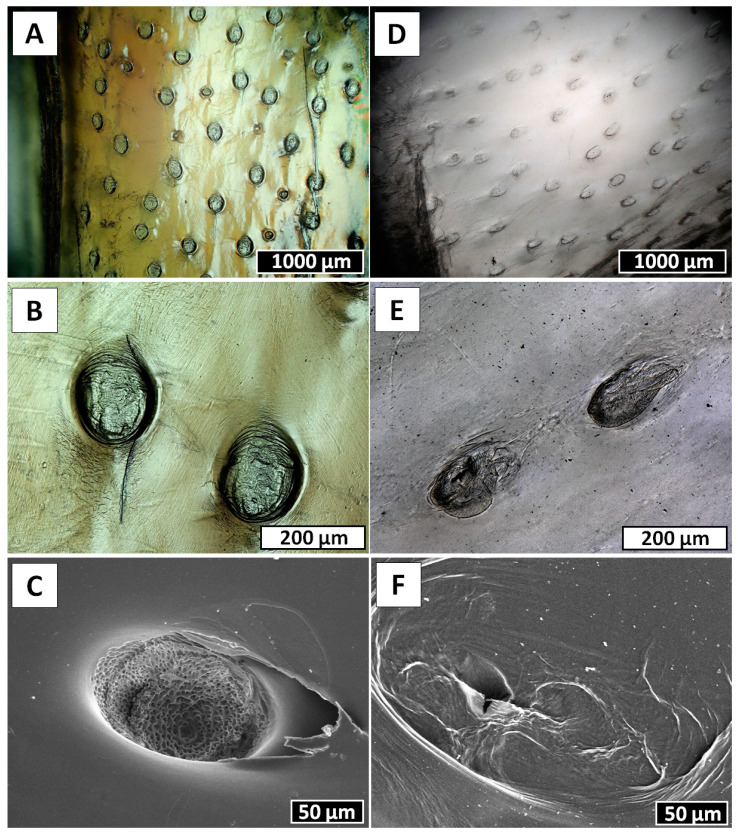
Surface morphology of the film-like, membranous structure, that was rejected from the *Cirrhipathes* sp. coral stem after 3 h of electrolysis (see Figure 4 and Figure 5), continues to be structurally changed following 6 h of electrolysis. Disappearance of spines becomes well visible using digital (**A**,**B**,**D**,**E**) as well as SEM (**C**,**F**) microscopy (see also Figure 7).

**Figure 7:**
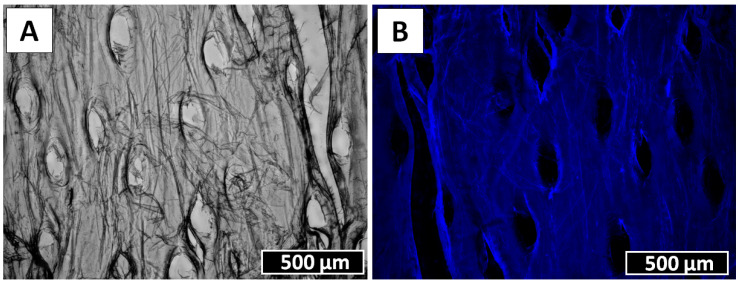
*Cirrhipathes* sp. sample after 12 h of electrolysis (**A**) and after Calcofluor white staining for preliminary chitin identification (**B**) (Light exposure time 1/500 s).

Following 6 h of electrolysis, both digital (Figure 6A,B,D,E) and scanning electron microscopy (Figure 6C,F) images showed defined structural changes. Spines partially disappeared, and only spongy and nanoporous structures (Figure 6C) became visible at the sites of their previous localization, though at some sites these structures also disappeared (Figure 6F). We suggest that these spines are composed of proteins and not of chitin, which is well recognized as a biological material with high resistance to alkaline treatment [4,5,6]. After 12 h of electrolysis, the nanoporous structures disappeared completely (Figure 7). Thus, we obtained a membranous organic matrix, with regular pores up to 100 µm large. Calcofluor white staining of this matter (Figure 7B) allows us to assume, with a high probability, the chitinous nature of the matrix, taking into account previously published results on chitin identification using this broadly applied technique [3,4,5,9,10,13,16,46,47,48,49,50,51,52,53,54,55,56,57,58].

For indisputable identification of chitin, we used infrared spectroscopy (ATR FT-IR), the chitinase digestion test, and ESI-MS-based analytics as represented below.

**Figure 8:**
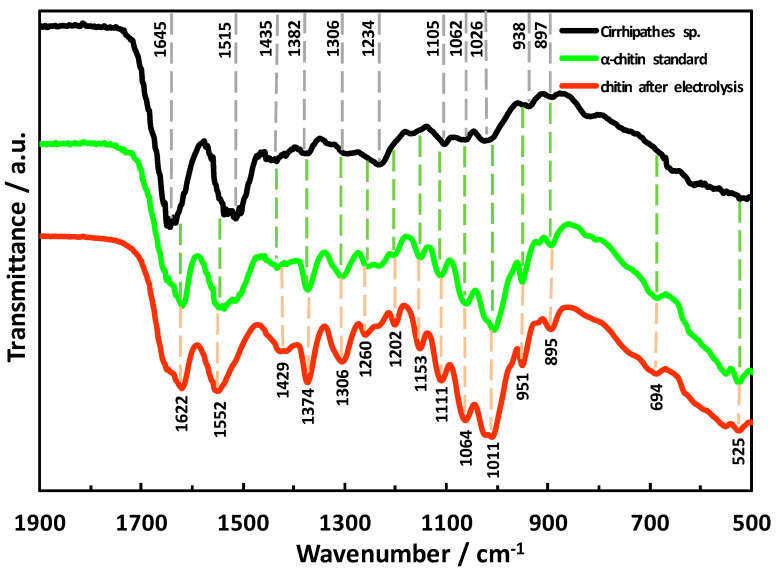
ATR-FTIR spectra of *Cirrhipathes* sp. sample (black line), α-chitin standard (green line) and electrochemically isolated chitinous scaffold (red line) in the region of 1900–500 cm^−1^.

ATR-FTIR spectroscopy was used to identify the functional groups typical for chitin in the investigated samples. Spectra, obtained for the naturally occurring rod of *Cirrhipathes* sp., the α-chitin standard, and a chitinous membrane-like scaffold (Figure 7A) which was isolated after full electrolysis treatment, are shown in Figure 8. The spectrum of the analyzed coral skeleton fragment (Figure 1B) shows bands which are similar to those previously reported for antipatharians by other authors [30,31]. Since the chemical composition of the *Cirrhipathes* sp.’s skeleton is highly diverse and complex (scleroproteins, lipids, diphenols and polysaccharides), most of the characteristic bands within the spectrum are overlapped by each other. Despite this, the characteristic bands for α-chitin, such as amide I (carbonyl stretching vibrations of *N*-acetyl groups) at 1645 cm^−1^, amide II (*ν*N–H and *ν*C–N) at 1531–1510 cm^−1^ and amide III (νC–N and δN–H) at 1306 cm^−1^, visible in the analyzed spectrum (Figure 8, black line), are sufficient to confirm the presence of chitin [6,59] within the sample under study. IR analysis of the isolated chitinous scaffold spectrum (Figure 8, red line) indicated that the characteristic band at 895 cm^−1^ (C–H deformation of the β-glycosidic bond, as well as the C–O–C bridge) suggests the occurrence of α-chitin in the sample (for comparison, 890 cm^−1^ for β-chitin) [4,6,33,45,46,47,48]. Moreover, the wavelengths of all other characteristic bands in this specimen are nearly identical to the α-chitin standard spectrum (Figure 8, green line), which additionally proves the presence of chitin in the α form in the electrochemically isolated organic matter. No presence of chitosan, as a possible product of the chitin de-acetylation under electrolysis conditions used here, has been confirmed using infrared spectroscopy.

**Figure 9:**
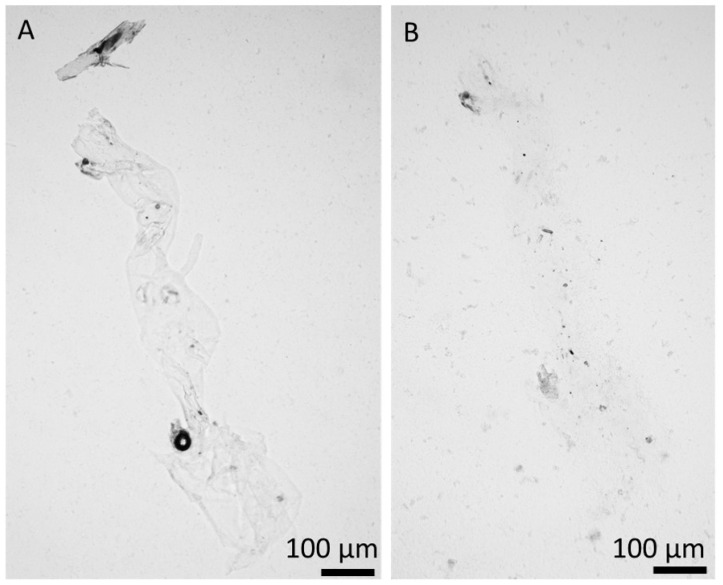
Chitinase digestion of electrochemically extracted chitin isolated from *Cirrhipathes* sp. at room temperature (light microscopic images). (**A**) Initial stage; (**B**) after 12 h of chitinase treatment.

Determination of *N*-acetylglucosamine (GlcNAc) is a key step for chitin identification in biological materials of unknown origin. To quantify chitin in the specimens of the membranaceous matter, isolated from *Cirrhipathes* sp. after 12 h of electrolysis, we measured the quantity of GlcNAc, released by chitinases using a classical Morgan–Elson colorimetric assay, which, owing to its specificity, is recognized as the most reliable method for the identification of alkali-insoluble chitin [4,5,6,47,48]. We detected 875.3 + 0.5 µg of *N*-acetylglucosamine per mg of depigmented skeleton of *Cirrhipathes* sp. Furthermore, the chitinase digestion test, based on the observation of the enzymatic dissolution of the purified, pigments- and proteins-free black coral organic matrix by light microscopy, confirmed the presence of pure chitin (Figure 9). Previously, we have shown that chemically impure chitin cannot be digested in chitinase solution [3,13].

**Figure 10:**
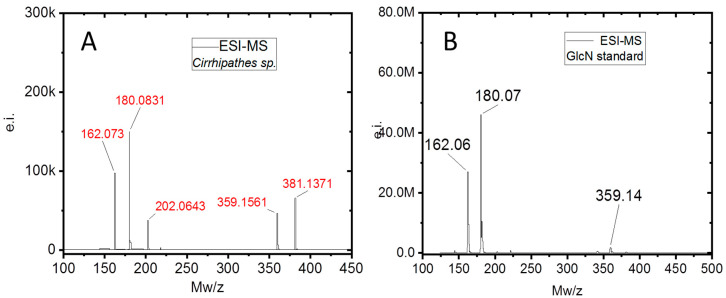
Electrospray-ionization mass spectroscopy (ESI-MS) characterization. (**A**) *Cirrhipathes* sp. after acid hydrolysis. (**B**) ESI-MS spectrum of GlcN standard reference.

It is well recognized that the d-glucosamine (GlcN) signals in the mass spectrum of hydrolyzed (6M HCl) biological samples reveal the presence of chitin. This method was utilized previously for chitin identification in diverse chitin-producing organisms [9,13,47,48], including heavy mineralized fossil specimens [46]. The acid hydrolysis of the *Cirrhipathes* sp. sample (see Figure 7A) revealed five main signals (Figure 10). The signals with *m*/*z* = 162 and 180 correspond to the [M − H_2_O + H^+^], [M + H^+^], while species with signal *m*/*z* = 359 correspond to proton-bound non covalent GlcN dimmer [**2**M + H^+^], which is common for ESI-MS spectra of amino monosaccharides. The signals with *m*/*z* = 202 and 381 correspond to the same species in which the hydrogen ion is substituted on sodium [M + Na^+^] and [2M + Na^+^], respectively, which is very common for the spectra of natural samples. Together, these results prove that the membrane-like, alkali-resistant biological material isolated from *Cirrhipathes* sp. contains chitin biopolymer.

**Figure 11:**
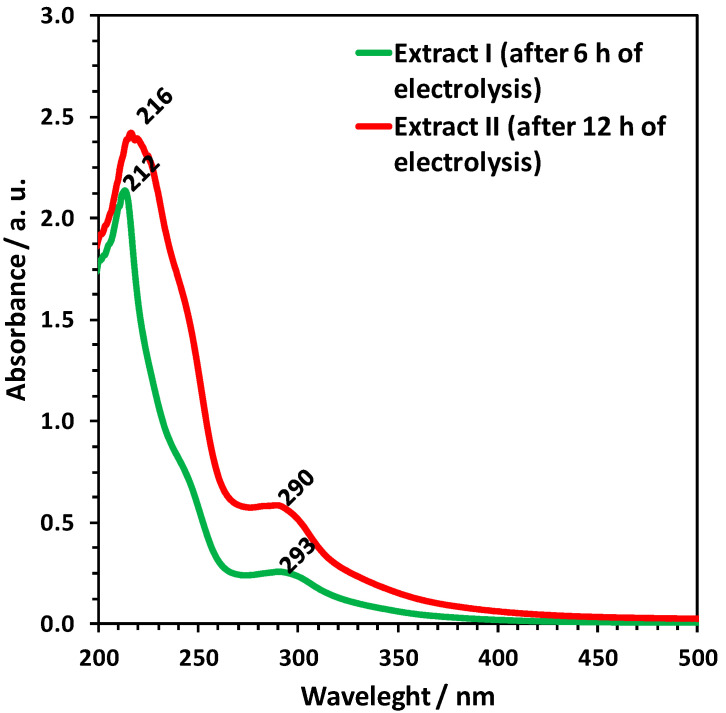
UV-visible absorption spectra of catholyte extracts isolated from the specimens of *Cirrhipathes* sp., following electrolysis for 6 h (green line) and 12 h (red line), suggest the catechol-like nature of the pigments.

Chitin was not the only product that was isolated from *Cirrhipathes* sp. using the electrochemical procedure represented in our study. Electrochemically mediated alkalization of the medium leads to the extraction of black pigment from the chitinous matter. Figure 11 shows the UV-visible spectra of the extracts obtained from the cathode chamber, after 6 h (extract I) and 12 h (extract II) of electrolytic treatment. According to data in the literature [60], both spectra show bands characteristic for polyphenols, most likely catechol derivatives, due to the peaks at 212 nm and 293 nm (Figure 11; extract I—green line) and 216 nm and 290 nm (extract II—red line). The diphenol trace within the extract samples could have resulted from the decomposition of the antipathin–chitin structural complex, as well as the reduction of 3,4-dihydroxybenzaldehyde (DOBAL) and 3-(3,4-dihydroxyphenyl)-l-alanine (DOPA) compounds [32,61]. We suggest that the presence of catechol-related compounds in the skeleton of the black coral *Cirrhipathes* sp. affects its fine, solid structure, ultimately resulting in the easier erosion of this biocomposite-based construct by NaOH. Similar results were reported for chitin isolated from beetle larva, where derivatives of catechol in its cuticle inhibited chitin crystallinity and led to the amorphous chitin structure [62].

## 3. Discussion

Black corals (Hexacorallia: Antipatharia) are a taxonomic group that appear worldwide in all the oceans and exhibit a wide depth distribution, ranging between coastal shallows and abyssal depths [63,64,65]. To date, the order Antipatharia comprises about 247 valid species, and they are most abundant in tropical and subtropical seas, in deep waters (≥50 m) beneath the photic zone [63,66,67]. For this reason, since most species are found below the depth limits of conventional SCUBA diving, very little is known about the basic biology and ecology of black corals. Despite this, all these species are characterized as having exclusively colonial habitus, with generally slow growth rates depending on the environment and high longevity, varying from decades to millennia [68,69,70,71,72]. Antipatharian colonies have various morphologies, either unbranched in the form of a whip or branched into a bush or a fan, with maximum sizes among species ranging from a few centimeters to many meters [73,74]. Moreover, to ensure stiffness and high mechanical strength, which are necessary to withstand high hydrostatic pressure and turbulent oceanic currents, the skeletons of antipatharians (Figure 1) usually have a layered scleroproteinaceous structure, strengthened by an inner chitinous scaffold [33,75]. The general morphology of their skeletons has been described as a laminated composite (Figure 2), where chitinous fibrils play a crucial role in terms of the growth support and elasticity of the entire colony. In addition, the internal structure of spines is believed to represent a significant reinforcing effect in the architecture of black coral skeletons, where the forces of the torsion imposed by currents are released [32]. This is particularly important in whip black corals, such as those belonging to the genera *Cirrhipathes*, *Stichopathes* and *Pseudocirrhipathes*, forming dense forests in the sites with the highest currents [76]. However, the dominant fraction within black coral skeletons is represented by a non-fibrillar formation, composed mostly of a halogen-containing scleroprotein and chitin [77]. This compound, known as antipathin, is exclusive to this taxon, and has unequalled thermal and mechanical stability which ensures the stiffness of the coral skeleton. Moreover, the chitin–antipathin based composite shows a unique combination of flexibility and hardness that provides better resistance to stress factors in a marine environment than inorganic structural materials [14]. Beside this, the chemical arrangement of the black coral skeleton also contains proteins, lipids and diphenols, and the chitin content within is estimated to constitute between 6% and 18% of the total organism mass (which is a considerable amount for marine invertebrates). Therefore, black corals can be considered as a potential source of chitinous scaffolds [78].

The finding of chitin within the skeletal structures of black coral *Cirrhipathes* sp. is important for gaining a better understanding of the structural biology of these organisms. Of course, black coral chitin cannot be used for practical application on a large scale, as is the case with crustacean or sea sponge chitin. However, this type of chitin is an interesting biomaterial in terms of its inspirational potential for biomimetics and material science. For example, the development of new chitin–catechol composite materials has an intriguing potential in biomedicine and technology. The first attempts in this direction have been recently made and patented (EP2778179A2 Chitosan and/or chitin composite having reinforced physical properties and use thereof. 2015). Definitively, more detailed studies on the chemistry and biosynthesis of naturally occurring chitin–polyphenol composites should be carried out in the near future. Chitin matrices of this type, with regularly located micropores, can serve as model systems for biomimetic studies into the creation of chitin-based membranes. An equally promising direction may be the creation of chitin membranes modified with polyphenolic compounds, which have antibacterial properties. The study of such membranes for the treatment of burns and other wounds seems to be in demand.

## 4. Materials and Methods 

### 4.1. Biological Samples and Chemicals 

*Cirrhipathes* sp. dry sample was purchased from INTIB GmbH (Freiberg, Germany). Sodium sulfate (Na_2_SO_4_, ≥99.7%), purchased from VWR (Darmstadt, Germany), was used for the preparation of aqueous electrolyte solution. Sodium hydroxide (NaOH, ≥99.0%), purchased from VWR (Darmstadt, Germany), was utilized as substrate to prepare an extracting solution. Distilled water was used to prepare all aqueous solutions. 

### 4.2. Electrolytic Cell Setup 

**Figure 12:**
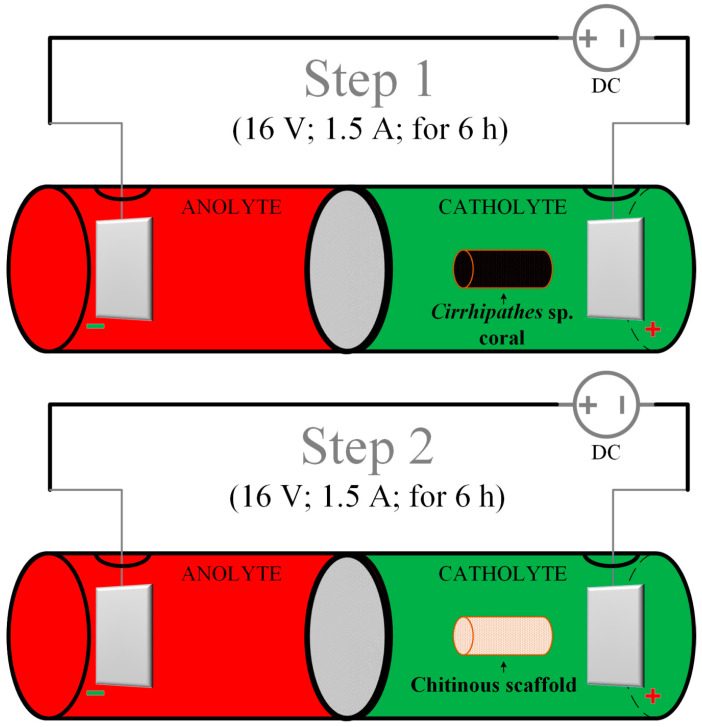
Schematic illustration of the experimental setup for all steps of the electrochemically-assisted isolation of *Cirrhipathes* sp. chitin [43].

The schematic illustration of the experimental system for the electrochemically-assisted isolation of chitin is shown in Figure 12. The CEM (cation exchange membrane) electrolyzer consisted of two cylindrical poly(propylene) chambers (50 mL each) separated by a cellulose membrane made from filter paper (75 g cm^−2^, ChemLand, Poland) and sealed with parafilm (Bemis Company Inc., Neenah, WI, USA). Electrodes (cathode and anode) were made of platinum sheets (effective area: 2.2 cm^2^). Distance between both electrodes was kept at about 10.0 cm and they were connected with DC power supply VoltCraft PS2043D (Conrad Electronic International GmbH & Co., Wels, Austria) by platinum wire current collectors. 1.9 M sodium sulfate aqueous solution with an initial temperature of 40 °C was utilized as anolyte as well as catholyte.

### 4.3. Electrochemically-Assisted Isolation of Chitin 

A novel electrochemically-assisted method of chitinous scaffold isolation from *Cirrhipathes* sp. was performed in two main steps (see Figure 12). In both stages, the sample was treated in the catholyte solution, and anolyte treatment (low pH) was not necessary to remove proteins, lipids and pigments. It should be noted that the initial concentration of the electrolyte (Na_2_SO_4_) for every step was 1.9 mol L^−1^ and the starting temperature for both anolyte and catholyte was 40 °C. 

**Pretreatment:** A 0.3 g piece was cut from the *Cirrhipathes* sp. sample and rinsed repeatedly with distilled water (25 °C) in order to get rid of the major solid impurities and water-soluble salts of marine origin. 

**Step 1:** The first part of the electro-alkali treatment was performed in the cathode chamber for 6 h (16 V, 1.5 A, 70 °C). High pH (up to 12.5) of the catholyte caused complete lysis of corals cells and degradation of lipids and proteins, which resulted in the removal of soft tissues from interlayer spaces of the chitinous skeleton. Moreover, this effect was followed by partial depigmentation and possible desilicification of the sample. Remaining chitinous skeleton was in the form of a light brown cell-free layered tube.

**Step 2:** In order to complete the depigmentation and deproteinization, the exchange of the electrolyte was required. With fresh 1.9 M Na_2_SO_4_ solution, the process was further carried out in the cathode chamber for 6 h (16 V, 1.5 A, 70 °C). Free access of the catholyte solution to the coral skeleton, along with high pH (up to 12.5), resulted in complete dissolution of pigments and residual proteins. After treatment, the remaining sample in the form of a colorless scaffold was extensively rinsed using distilled water up to neutral pH, and stored in ethanol absolute (4 °C).

### 4.4. Calcofluor White Staining 

The Calcofluor white staining (CFW) (Fluorescent Brightener M2R, Sigma-Aldrich, St. Louis, MO, USA) was used to confirm the presence of chitin in the sponge skeleton several times (see [5,6,7,8]). For the staining process, 30 µL of a solution containing 10 g glycerin and 10 g NaOH in 90 mL of water was applied. After a minute, the CFW was added, and the investigated material was incubated in staining solution for 6 h without light at 25 °C. Then, the sample was washed with distilled water to eliminate the unattached stain, dried at 25 °C and analyzed using fluorescent microscopy. On binding to polysaccharides containing β-glycosidic bonds (such as chitin), this fluorochrome secretes bright blue light under UV excitation even with a very short light exposure time.

### 4.5. Chitinase Digestion Test 

The fragment of isolated chitinous scaffold from *Cirrhipathes* sp. was treated with Yatalase enzyme solution (pH 6.5) [58]. The treatment was carried out for 6 h at 37 °C. The progress of digestion was observed under light microscopy using BZ-9000 microscope (Keyence, Osaka, Japan). 

### 4.6. Attenuated Total Reflectance Fourier Transform Infrared Spectroscopy 

Attenuated Total Reflectance Fourier Transform Infrared Spectroscopy (ATR-FTIR) was used for the qualitative characterization and identification of the isolated materials. The samples were analyzed by Nicolet 210c spectrometer (Thermo Fisher Scientific, Waltham, MA, USA).

### 4.7. Estimation of N-acetyl-d-glucosamine (NAG) Contents

The Morgan–Elson assay was used to quantify the *N*-acetyl-d-glucosamine released after chitinase treatment, as described previously [79]. Purified and dried *Cirrhipathes* sp. samples (6 mg) were pulverized to fine powder in an agate mortar. The samples were suspended in 400 mL of 0.2 M phosphate buffer at pH 6.5. A positive control was prepared by solubilizing 0.3% colloidal chitin (INTIB GmbH, Freiberg, Germany) in the same buffer. Equal amounts of 1 mg/mL from three chitinases (EC 3.2.1.14 and EC 3.2.1.30)—*N*-acetyl-d-glucosaminidase from *Trichoderma viride* (Sigma, No. C-8241), and two poly (1,4-α-[2-acetamido-2-deoxy-d-glucoside]) glycanohydrolases from *Serratia marcescens* (Sigma, No. C-7809), and *Streptomyces griseus* (Sigma, No. C-6137)—were suspended in 100 mM sodium phosphate buffer at pH 6.0. Digestion was initiated by mixing 400 mL of the sample and 400 mL of the chitinase mix. Incubation was performed at 37 °C and stopped after 114 h by adding 400 mL of 1% NaOH, followed by boiling for 5 min. The vessels were centrifuged at 7000 rpm for 5 min and the purified reducing sugars were used for 3,5-dinitrosalicylic acid assay (DNS) [47,48]. For this purpose, 250 mL of the supernatants and 250 mL of 1% DNS were dissolved in a solution containing 30% sodium potassium tartrate in 0.4 M NaOH. The reagents were mixed and incubated for 5 min in a boiling water bath. Thereafter, the absorbance at 540 nm was recorded using a Tecan Spectrafluor Plus Instrument (Mannedorf/Zurich, Switzerland). Data were interpolated using a standard curve prepared with a series of dilutions (0–3.0 mM) of *N*-acetyl-d-glucosamine (Sigma, No. A-8625) and DNS. A sample, which contained chitinase solution without substrate, was used as a control.

### 4.8. Electrospray Ionization Mass Spectrometry (ESI-MS) 

Specimens obtained after electrochemical isolation in the final step (Figure 7A) were hydrolyzed in 6 M HCl for 24 h at 50 °C. Following the HCl hydrolysis, the samples were filtrated with 0.4 µm filter and freeze-dried in order to remove excess HCl. The remaining solid was dissolved in water for ESI-MS analysis. As standard, d-glucosamine was purchased from Sigma-Aldrich (Taufkirchen, Germany). The ESI-MS analytical measurements were performed using Agilent Technologies 6230 TOF LC/MS spectrometer (Applied Biosystems, Santa Clara, CA, USA). Nitrogen was used as the nebulizing and desolvation gas. Graphs were generated using Origin 8.5 for PC (Originlab Corporation, Northampton, MA, USA).

### 4.9. UV-VIS Spectroscopy

To conduct UV-VIS Spectroscopy, 0.5 mg of pigments, electrochemically isolated from *Cirrhipathes* sp. (Figure 1), were dissolved in 1 mL of 0.1 M KOH. The spectra were measured by JASCO V-750 spectrometer, in the wavelength range of 200 to 800 nm, which was operated at a resolution of 5 nm using a quartz cuvette with path length of 1 cm (quartz suprasil, Hellma Analytics, Müllheim, Germany). 

### 4.10. Scanning Electron Microscopy (SEM)

The specimens were fixed on an aluminum sample holder with conductive carbon adhesive tabs and were sputtered with platinum for 15 s at a distance of 30 mm by an Edwards S150B sputter coater. The scanning electron micrographs were observed using a high-resolution Hitachi S-4700-II (Hitachi, Ltd., Tokyo, Japan) equipped with a cold field emission gun. 

## 5. Conclusions

In the present work, we utilized the in-situ electrolysis of a 1.9 M Na_2_SO_4_ aqueous solution in the CEM of an electrolyzer as the method for isolating chitinous scaffolds from *Cirrhipathes* sp. black coral. The final results of the electrochemically-assisted isolation of chitin were a colorless, membrane-like film, and catechol-based extracts. The digital light and scanning electron microscopy investigations of this final product revealed that, despite the highly alkaline environment of the catholyte and the destruction of proteins within the coral’s skeleton, the general spatial structure of the sample preserved its original membranous formation, with regular pores up to 100 µm large. Further characterization of the isolated sample with various techniques proved that a pure chitinous scaffold can be obtained via the application of the described method. Moreover, as the ATR-FTIR spectroscopy analysis showed, the electrochemically-supported isolation process does not cause a chitin–chitosan transformation, and the obtained scaffold was fully α-chitin. All these features, boosted additionally by the advantages of the electrolysis method (i.e., reduction of time treatment and amount of chemicals used), show that our method can be considered as an alternative to the standard chemical chitin extraction process. Thus, without doubt further development of the electrochemical isolation of chitin from marine sources should be carried out in the near future.

## References

[1] Rinaudo M. (2006). Chitin and chitosan: Properties and applications. Prog. Polym. Sci..

[2] Rahman A., Halfar J. (2014). First evidence of chitin in calcified coralline algae: New insights into the calcification process of Clathromorphum compactum. Sci. Rep..

[3] Brunner E., Ehrlich H., Schupp P., Hedrich R., Hunoldt S., Kammer M., Machill S., Paasch S., Bazhenov V., Kurek D. (2009). Chitin-based scaffolds are an integral part of the skeleton of the marine demosponge Ianthella basta. J. Struct. Biol..

[4] Ehrlich H., Maldonado M., Spindler K.-D., Eckert C., Hanke T., Born R., Goebel C., Simon P., Heinemann S., Worch H. (2007). First evidence of chitin as a component of the skeletal fibers of marine sponges. Part I. Verongidae (demospongia: Porifera). J. Exp. Zoöl. Part B: Mol. Dev. Evol..

[5] Ehrlich H., Krautter M., Hanke T., Simon P., Knieb C., Heinemann S., Worch H. (2007). First evidence of the presence of chitin in skeletons of marine sponges. Part II. Glass sponges (Hexactinellida: Porifera). J. Exp. Zoöl. Part B: Mol. Dev. Evol..

[6] Ehrlich H., Ilan M., Maldonado M., Muricy G., Bavestrello G., Kljajic Z., Carballo J.L., Schiaparelli S., Ereskovsky A., Schupp P. (2010). Three-dimensional chitin-based scaffolds from Verongida sponges (Demospongiae: Porifera). Part I. Isolation and identification of chitin. Int. J. Biol. Macromol..

[7] Liu S., Sun J., Yu L., Zhang C., Bi J., Zhu F., Qu M., Jiang C., Yang Q. (2012). Extraction and Characterization of Chitin from the Beetle Holotrichia parallela Motschulsky. Molecules.

[8] Kaya M., Seyyar O., Baran T., Erdogan S., Kar M. (2014). A physicochemical characterization of fully acetylated chitin structure isolated from two spider species: With new surface morphology. Int. J. Biol. Macromol..

[9] Machałowski T., Wysokowski M., Tsurkan M., Galli R., Schimpf C., Rafaja D., Brendler E., Viehweger C., Żółtowska-Aksamitowska S., Petrenko I. (2019). Spider Chitin: An Ultrafast Microwave-Assisted Method for Chitin Isolation from Caribena versicolor Spider Molt Cuticle. Molecules.

[10] Machałowski T., Wysokowski M., Żółtowska-Aksamitowska S., Bechmann N., Binnewerg B., Schubert M., Guan K., Bornstein S.R., Czaczyk K., Pokrovsky O. (2019). Spider Chitin. The biomimetic potential and applications of Caribena versicolor tubular chitin. Carbohydr. Polym..

[11] Tolesa L.D., Gupta B.S., Lee M.-J. (2019). Chitin and chitosan production from shrimp shells using ammonium-based ionic liquids. Int. J. Biol. Macromol..

[12] Mohan K., Ravichandran S., Muralisankar T., Uthayakumar V., Chandirasekar R., Rajeevgandhi C., Rajan D.K., Seedevi P. (2019). Extraction and characterization of chitin from sea snail Conus inscriptus (Reeve, 1843). Int. J. Biol. Macromol..

[13] Ehrlich H., Bazhenov V., Debitus C., De Voogd N., Galli R., Tsurkan M., Wysokowski M., Meissner H., Bulut E., Kaya M. (2017). Isolation and identification of chitin from heavy mineralized skeleton of Suberea clavata (Verongida: Demospongiae: Porifera) marine demosponge. Int. J. Biol. Macromol..

[14] Ehrlich H. (2019). Marine Biological Materials of Invertebrate Origin.

[15] Klinger C., Żółtowska-Aksamitowska S., Wysokowski M., Tsurkan M., Galli R., Petrenko I., Machałowski T., Ereskovsky A.V., Martinovic R., Muzychka L. (2019). Express Method for Isolation of Ready-to-Use 3D Chitin Scaffolds from Aplysina archeri (Aplysineidae: Verongiida) Demosponge. Mar. Drugs.

[16] Nowacki K., Stępniak I., Machałowski T., Wysokowski M., Petrenko I., Schimpf C., Rafaja D., Langer E., Richter A., Ziętek J. (2020). Electrochemical method for isolation of chitinous 3D scaffolds from cultivated Aplysina aerophoba marine demosponge and its biomimetic application. Appl. Phys. A.

[17] Soon C.Y., Tee Y.B., Tan C.H., Rosnita A.T., Khalina A. (2018). Extraction and physicochemical characterization of chitin and chitosan from Zophobas morio larvae in varying sodium hydroxide concentration. Int. J. Biol. Macromol..

[18] Younes I., Rinaudo M. (2015). Chitin and Chitosan Preparation from Marine Sources. Structure, Properties and Applications. Mar. Drugs.

[19] Ehrlich H., Shaala L.A., Youssef D.T.A., Żółtowska-Aksamitowska S., Tsurkan M., Galli R., Meissner H., Wysokowski M., Petrenko I., Tabachnick K.R. (2018). Discovery of chitin in skeletons of non-verongiid Red Sea demosponges. PLoS ONE.

[20] Percot A., Viton C., Domard A. (2003). Optimization of Chitin Extraction from Shrimp Shells. Biomacromolecules.

[21] Khanafari A., Marandi R., Sanatei S. (2008). Recovery of chitin and chitosan from shrimp waste by chemical and microbal methods. Iran. J. Environ. Health Sci. Eng..

[22] Knidri H., Dahmani J., Addaou A., Laajeb A., Lahsini A. (2019). Rapid and efficient extraction of chitin and chitosan for scale-up production: Effect of process parameters on deacetylation degree and molecular weight. Int. J. Biol. Macromol..

[23] Kuprina E.E., Maslova G.V., Bachische E.V. Electrochemical method for obtaining water-soluble oligomers of chitin in the presence of NaCl. Proceedings of the IXth International Conference: Modern Perspectives in Chitin and Chitosan Studies.

[24] Feng M., Lu X., Zhang J., Li Y., Shi C., Lu L., Zhang S. (2019). Direct conversion of shrimp shells to O-acylated chitin with antibacterial and anti-tumor effects by natural deep eutectic solvents. Green Chem..

[25] Kuprina E.E., Vodolazhskaya S.V., Nyanikova G.G., Timofeeva K.G. Development of technology for obtaining biologically active chitin sorbents based on the electrochemical conversion of crustaceans. Proceedings of the VIth International Conference: New Achievements in Study of Chitin and Chitosan.

[26] Kuprina E.E., Timofeeva K.G., Kozlova I., Pimenov A. Electrochemical method extracting sorbitol from chitin-containing raw material with strengthened anitimicrobal properties. Proceedings of the VIIth International Conference: Modern Perspectives in Chitin and Chitosan Studies.

[27] Kuprina E.E., Timofeeva K.G., Krasavtsev V.E., Boykov I.O.A. Experimental producing unit for getting chitin-mineral complex “chizitel” by electrochemical method. Proceedings of the VIIIth International Conference: Modern Perspectives in Chitin and Chitosan Studies.

[28] Kuprina E.É., Timofeeva K.G., Vodolazhskaya S.V. (2002). Electrochemical Preparation of Chitin Materials. Russ. J. Appl. Chem..

[29] Tennakone K. (1989). Hydrogen from brine electrolysis: A new approach. Int. J. Hydrogen Energy.

[30] La Rosa B.J.-D., Ardisson P.-L., Azamar-Barrios J., Quintana P., Alvarado-Gil J.J. (2007). Optical, thermal, and structural characterization of the sclerotized skeleton of two antipatharian coral species. Mater. Sci. Eng. C.

[31] Nowak D., Florek M., Nowak J., Kwiatek W.M., Lekki J., Chevallier P., Hacura A., Wrzalik R., Ben-Nissan B., Van Grieken R. (2009). Morphology and the chemical make-up of the inorganic components of black corals. Mater. Sci. Eng. C.

[32] Kim K., Goldberg W.M., Taylor G.T. (1992). Architectural and Mechanical Properties of the Black Coral Skeleton (Coelenterata: Antipatharia): A Comparison of Two Species. Biol. Bull..

[33] Bo M., Bavestrello G., Kurek D., Paasch S., Brunner E., Born R., Galli R., Stelling A.L., Sivkov V.N., Petrova O.V. (2012). Isolation and identification of chitin in black coral Paranthipates larix (Anthozoa: Cnidaria). Int. J. Biol. Macromol..

[34] Kuprina E.E., Krasavtsev V., Kozlova I., Vodolazhskaya S., Bogeruk A., Ezhov V. Electrochemical method of chitinous products with enhanced ecology rehabilitation ability. Proceedings of the Vth International Conference: New Prospects in Study of Chitin and Chitosan.

[35] Pletcher D., Walsh F.C. (1993). Industrial Electrochemistry.

[36] Strathmann H. (2004). Ion-Exchange Membrane Separation Processes. Membr. Sci. Tech..

[37] Savari S., Sachdeva S., Kumar A. (2008). Electrolysis of sodium chloride using composite poly(styrene-co-divinylbenzene) cation exchange membranes. J. Membr. Sci..

[38] Zeppilli M., Lai A., Villano M., Majone M. (2016). Anion vs. cation exchange membrane strongly affect mechanisms and yield of CO_2_ fixation in a microbial electrolysis cell. Chem. Eng. J..

[39] Holze R., Ahn J. (1992). Advances in the use of perfluorinated cation exchange membranes in integrated water electrolysis and hydrogen/oxygen fuel cell systems. J. Membr. Sci..

[40] Park J.E., Kang S.Y., Oh S.-H., Kim J.K., Lim M.S., Ahn C.-Y., Cho Y.-H., Sung Y.-E. (2019). High-performance anion-exchange membrane water electrolysis. Electrochim. Acta.

[41] Salvatore D.A., Weekes D.M., He J., Dettelbach K.E., Li Y.C., Mallouk T.E., Berlinguette C.P. (2017). Electrolysis of Gaseous CO_2_ to CO in a Flow Cell with a Bipolar Membrane. ACS Energy Lett..

[42] Pisarska B., Wicher I., Dylewski R. (2004). Studies on the parameters for membrane-electrolysis conversion of sodium sulfate solutions. Przemysł Chem..

[43] Holze S., Jörissen J., Fischer C., Kalvelage H. (1994). Hydrogen consuming anodes for energy saving in sodium sulphate electrolysis. Chem. Eng. Technol..

[44] Jörissen J., Simmrock K.H. (1991). The behavior of ion exchange membranes in electrolysis and electrodialysis of sodium sulfate. J. Appl. Electrochem..

[45] Kumirska J., Czerwicka M.T., Kaczynski Z., Bychowska A., Brzozowski K., Thöming J., Stepnowski P. (2010). Application of Spectroscopic Methods for Structural Analysis of Chitin and Chitosan. Mar. Drugs.

[46] Ehrlich H., Rigby J.K., Botting J.P., Tsurkan M., Werner C., Schwille P., Petrásek Z., Pisera A., Simon P., Sivkov V.N. (2013). Discovery of 505-million-year old chitin in the basal demosponge Vauxia gracilenta. Sci. Rep..

[47] Ehrlich H., Kaluzhnaya O.V., Tsurkan M., Ereskovsky A.V., Tabachnick K.R., Ilan M., Stelling A., Galli R., Petrova O.V., Nekipelov S.V. (2013). First report on chitinous holdfast in sponges (Porifera). Proc. R. Soc. B.

[48] Ehrlich H., Kaluzhnaya O.V., Brunner E., Tsurkan M., Ereskovsky A.V., Ilan M., Tabachnick K.R., Bazhenov V., Paasch S., Kammer M. (2013). Identification and first insights into the structure and biosynthesis of chitin from the freshwater sponge Spongilla lacustris. J. Struct. Biol..

[49] Henriques B.S., Garcia E.S., Azambuja P., Genta F.A. (2020). Determination of Chitin Content in Insects: An Alternate Method Based on Calcofluor Staining. Front. Physiol..

[50] Denny G., Khanna R., Hornstra I., Kwatra S.G., Grossberg A.L. (2020). Rapid detection of fungal elements using calcofluor white and handheld ultraviolet illumination. J. Am. Acad. Dermatol..

[51] Connors M.J., Ehrlich H., Hog M., Godeffroy C., Araya S., Kallai I., Gazit D., Boyce M., Ortiz C. (2012). Three-dimensional structure of the shell plate assembly of the chiton Tonicella marmorea and its biomechanical consequences. J. Struct. Biol..

[52] Wysokowski M., Motylenko M., Walter J., Lota G., Wojciechowski J., Stöcker H., Galli R., Stelling A.L., Himcinschi C., Niederschlag E. (2014). Synthesis of nanostructured chitin–hematite composites under extreme biomimetic conditions. RSC Adv..

[53] Żółtowska-Aksamitowska S., Tsurkan M., Lim S., Meissner H., Tabachnick K., Shaala L.A., Youssef D.T., Ivanenko V.N., Petrenko I., Wysokowski M. (2018). The demosponge Pseudoceratina purpurea as a new source of fibrous chitin. Int. J. Biol. Macromol..

[54] Żółtowska-Aksamitowska S., Shaala L.A., Youssef D.T.A., Elhady S.S., Tsurkan M., Petrenko I., Wysokowski M., Tabachnick K., Meißner H., Ivanenko V.N. (2018). First Report on Chitin in a Non-Verongiid Marine Demosponge: The Mycale euplectellioides Case. Mar. Drugs.

[55] Fromont J., Zoltowska-Aksamitowska S., Galli R., Meissner H., Erpenbeck D., Vacelet J., Diaz C., Tsurkan M.V., Petrenko I., Youssef D. (2019). New family and genus of a Dendrilla-like sponge with characters of Verongiida. Part II. Discovery of chitin in the skeleton of Ernstilla lacunosa. Zoologischer Anzeiger.

[56] Shaala L.A., Asfour H., Youssef D.T.A., Żółtowska-Aksamitowska S., Wysokowski M., Tsurkan M., Galli R., Meißner H., Petrenko I., Tabachnick K. (2019). New Source of 3D Chitin Scaffolds: The Red Sea Demosponge Pseudoceratina arabica (Pseudoceratinidae, Verongiida). Mar. Drugs.

[57] Schubert M., Binnewerg B., Voronkina A., Muzychka L., Wysokowski M., Petrenko I., Kovalchuk V., Tsurkan M., Martinovic R., Bechmann N. (2019). Naturally Prefabricated Marine Biomaterials: Isolation and Applications of Flat Chitinous 3D Scaffolds from Ianthella labyrinthus (Demospongiae: Verongiida). Int. J. Mol. Sci..

[58] Kovalchuk V., Voronkina A., Binnewerg B., Schubert M., Muzychka L., Wysokowski M., Tsurkan M., Bechmann N., Petrenko I., Fursov A. (2019). Naturally Drug-Loaded Chitin: Isolation and Applications. Mar. Drugs.

[59] Kaya M., Mujtaba M., Ehrlich H., Salaberria A.M., Baran T., Amemiya C.T., Galli R., Akyuz L., Sargin I., Labidi J. (2017). On chemistry of γ-chitin. Carbohydr. Polym..

[60] Pillar E.A., Zhou R., Guzman M. (2015). Heterogeneous Oxidation of Catechol. J. Phys. Chem. A.

[61] Holl S.M., Schaefer J., Goldberg W.M., Kramer K.J., Morgan T.D., Hopkins T.L. (1992). Comparison of black coral skeleton and insect cuticle by a combination of carbon-13 NMR and chemical analyses. Arch. Biochem. Biophys..

[62] Zhang M., Haga A., Sekiguchi H., Hirano S. (2000). Structure of insect chitin isolated from beetle larva cuticle and silkworm (*Bombyx mori*) pupa exuvia. Int. J. Biol. Macromol..

[63] Cairns S.D. (2007). Deep-water corals: An overview with special reference to diversity and distribution of deep-water scleractinian corals. Bull. Mar. Sci..

[64] Farfan G.A., Cordes E.E., Waller R.G., Decarlo T.M., Hansel C.M. (2018). Mineralogy of Deep-Sea Coral Aragonites as a Function of Aragonite Saturation State. Front. Mar. Sci..

[65] Brugler M.R., Opresko D.M., France S. (2013). The evolutionary history of the order Antipatharia (Cnidaria: Anthozoa: Hexacorallia) as inferred from mitochondrial and nuclear DNA: Implications for black coral taxonomy and systematics. Zool. J. Linn. Soc..

[66] Molodtsova T.N., Opresko D.M. (2017). Black corals (Anthozoa: Antipatharia) of the Clarion-Clipperton Fracture Zone. Mar. Biodivers..

[67] Daly M., Brugler M.R., Cartwright P., Collins A.G., Dawson M.N., Fautin D.G., France S., McFadden C.S., Opresko D.M., Rodriguez E. (2007). The phylum Cnidaria: A review of phylogenetic patterns and diversity 300 years after Linnaeus*. Zootaxa.

[68] Bo M., Di Camillo C., Addamo A.M., Valisano L., Bavestrello G. (2009). Growth strategies of whip black corals (Cnidaria: Antipatharia) in the Bunaken Marine Park (Celebes Sea, Indonesia). Mar. Biodivers. Rec..

[69] Bo M., Bavestrello G., Angiolillo M., Calcagnile L., Canese S., Cannas R., Cau A., D’Elia M., D’Oriano F., Follesa M.C. (2015). Persistence of Pristine Deep-Sea Coral Gardens in the Mediterranean Sea (SW Sardinia). PLoS ONE.

[70] Roark E.B., Guilderson T., Dunbar R., Ingram B. (2006). Radiocarbon-based ages and growth rates of Hawaiian deep-sea corals. Mar. Ecol. Prog. Ser..

[71] Prouty N., Roark E.B., Buster N., Ross S. (2011). Growth rate and age distribution of deep-sea black corals in the Gulf of Mexico. Mar. Ecol. Prog. Ser..

[72] Wagner D., Luck D.G., Toonen R.J. (2012). The Biology and Ecology of Black Corals (Cnidaria: Anthozoa: Hexacorallia: Antipatharia). Adv. Mar. Biol..

[73] Wagner D., Shuler A. (2017). The black coral fauna (Cnidaria: Antipatharia) of Bermuda with new records. Zootaxa.

[74] Gąsiorek P., Cordeiro R.T.S., Perez C.D. (2019). Black Corals (Anthozoa: Antipatharia) from the Southwestern Atlantic. Zootaxa.

[75] Goldberg W.M. (1978). Chemical changes accompanying maturation of the connective tissue skeletons of gorgonian and antipatharian corals. Mar. Biol..

[76] Tazioli S., Bo M., Boyer M., Rotinsulu H., Bavestrello G. (2007). Ecological observations of some common antipatharian corals in the marine park of Bunaken (North Sulawesi, Indonesia). Zool. Stud..

[77] Goldberg W.M., Hopkins T.L., Holl S.M., Schaefer J., Kramer K.J., Morgan T.D., Kim K. (1994). Chemical composition of the sclerotized black coral skeleton (Coelenterata: Antipatharia): A comparison of two species. Comp. Biochem. Physiol. Part B: Comp. Biochem..

[78] Goldberg W.M. (1991). Chemistry and structure of skeletal growth rings in the black coral Antipathes fiordensis (Cnidaria, Antipatharia). Hydrobiologia.

[79] Boden N., Sommer U., Spindler K.-D. (1985). Demonstration and characterization of chitinases in the Drosophila-K-cell Line. Insect Biochem..

